# Two Faced Janus of Quantum Nonlocality

**DOI:** 10.3390/e22030303

**Published:** 2020-03-06

**Authors:** Andrei Khrennikov

**Affiliations:** International Center for Mathematical Modeling in Physics and Cognitive Sciences, Linnaeus University, SE-351 95 Växjö, Sweden; Andrei.Khrennikov@lnu.se

**Keywords:** quantum nonlocality, Bell nonlocality, Einstein-Lüders nonlocality, projection postulate, state-transformation, probability conditioning, individual vs. statistical interpretations, quantum vs. classical superpositions, ontic-epistemic, spooky action vs. prediction at a distance

## Abstract

This paper is a new step towards understanding why “quantum nonlocality” is a misleading concept. Metaphorically speaking, “quantum nonlocality” is Janus faced. One face is an apparent nonlocality of the Lüders projection and another face is Bell nonlocality (a wrong conclusion that the violation of Bell type inequalities implies the existence of mysterious instantaneous influences between distant physical systems). According to the Lüders projection postulate, a quantum measurement performed on one of the two distant entangled physical systems modifies their compound quantum state instantaneously. Therefore, if the quantum state is considered to be an attribute of the individual physical system and if one assumes that experimental outcomes are produced in a perfectly random way, one quickly arrives at the contradiction. It is a primary source of speculations about a spooky action at a distance. Bell nonlocality as defined above was explained and rejected by several authors; thus, we concentrate in this paper on the apparent nonlocality of the Lüders projection. As already pointed out by Einstein, the quantum paradoxes disappear if one adopts the purely statistical interpretation of quantum mechanics (QM). In the statistical interpretation of QM, if probabilities are considered to be objective properties of random experiments we show that the Lüders projection corresponds to the passage from joint probabilities describing all set of data to some marginal conditional probabilities describing some particular subsets of data. If one adopts a subjective interpretation of probabilities, such as QBism, then the Lüders projection corresponds to standard Bayesian updating of the probabilities. The latter represents degrees of beliefs of local agents about outcomes of individual measurements which are placed or which will be placed at distant locations. In both approaches, probability-transformation does not happen in the physical space, but only in the information space. Thus, all speculations about spooky interactions or spooky predictions at a distance are simply misleading. Coming back to Bell nonlocality, we recall that in a recent paper we demonstrated, using exclusively the quantum formalism, that CHSH inequalities may be violated for some quantum states only because of the incompatibility of quantum observables and Bohr’s complementarity. Finally, we explain that our criticism of quantum nonlocality is in the spirit of Hertz-Boltzmann methodology of scientific theories.

## 1. Introduction

In the last few decades too many futile technical terms in the context of correlation measurements have been created which are not well defined. They evoke different associations in the discussion on entangled systems and these associations are sometimes rather confusing. As was emphasized in recent paper [1] (see also [2]), the notion “quantum nonlocality” is really misleading. One of the difficulties in struggling with nonlocality is that (as was pointed in [3]) the present situation is the real mess. Surprisingly, this mess-problem is ignored and it is commonly claimed that quantum physics is “nonlocal” (without specifying what this means concretely).

**Figure 1:**
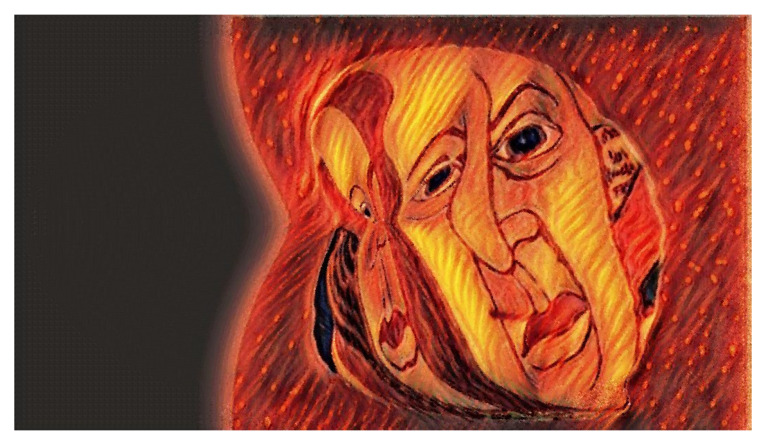
Two-faced Janus artwork of David S. Soriano (https://commons.wikimedia.org/wiki/File:Two-Faced_Janus_David_(S._Soriano)_Artwork.jpg).

Personally, I got the first signal that quantum nonlocality is Janus faced (see Figure 1) from the talk of Aspect at one of the Växjö conferences (see also his papers [4,5]). He started his talk not from the Bell inequality [6,7] and its violation (as could be expected), but with the projection postulate in Lüders’ form [8] and its nonlocal consequences. He pointed that the Lüders *projection nonlocality* is really counter-intuitive, and that to find a proper physical picture, one has to introduce hidden variables. From this viewpoint, quantum theory really cries for hidden variables! However, on this way, as is commonly accepted, one confronts the Bell inequality and proceeds towards *Bell nonlocality*. The latter is an unfounded claim that the violation of the Bell type inequalities may not be explained in a causally local way and that any subquantum hidden variable description of quantum correlations must invoke instantaneous influences at a distance (see, e.g., [9,10,11,12,13,14,15,16,17,18,19,20] for critical debating on this claim).

We remark that Aspect cleaned the well-known Einstein-Podolsky-Rosen (EPR) reasoning [21] from the elements of reality and statements with probability one. His presentation is essentially clearer than the original EPR-argument.

We remark that the projection postulate is often associated with von Neumann. It is common to speak about the von Neumann projection postulate. However, von Neumann [22] used it only for observables with non-degenerate spectra. Lüders suggested [8] to extend the applicability of the projection postulate to all observables (see also [23,24] for generalizations in the framework of the theory of quantum instruments).

In paper [1], I tried to decouple Bell nonlocality from quantum theory. The main message from [1] is that violation of the Bell type inequalities, predicted within the quantum formalism and tested experimentally, is the straightforward consequence of *local incompatibility of observables*. Thus, the Bell test can be interpreted as a very special test of the Bohr’s complementarity principle. Purely quantum treatment of the Bell type inequalities does not leave any place for nonlocal speculations. A similar conclusion can be found in works of De Muynck [25], Kupczynski [26], Griffiths [27], Boughn [28], Jung [29], Cetto, Valdes-Hernandez, and de la Pena [30].

The aim of the present paper is to destroy even the seed of Bell’s nonlocality—Lüders projection nonlocality that will be analyzed in great detail. We show that it is fictitious; this is the “nonlocality” of probability conditioning; it is identical to the “nonlocality” of classical probability conditioning.

We also emphasize the role of interpretations of a quantum state, individual (physical) vs. statistical (and its special version, *the Växjö interpretation* [31]).

In fact, I started writing this paper as a reply to the following comment of Johan Summhammer on my previous article [1]:

“I have looked at your ’Get rid of non locality…’ and I agree at the formal level, that you are only dealing with incompatible operators. But the empirical fact remains, that setting a measurement operator on site A has an instantaneous ’influence’ on site B, if you later calculate conditional statistics between the data of site A and site B. Additionally, this faster-than-light change of statistical correlation is already empirically proven. Sure, no transfer of information. But more than ’no influence.’ Which name would you assign to this phenomenon?”

Such often postulated instantaneous influence at a distance is not caused by a real or spooky interaction between the two distant observations. Actually, nothing else happens, but that our knowledge about the entangled quantum state, especially about the second photon, is increased by measurements on the first photon. The information about the outcome of the first detection process distinctly restricts the probabilities of the possible outcomes of the second measurement process. This reduction of possibilities is a mental process, not a physical one. It can be described by an update of our knowledge. If the statistical interpretation of quantum mechanics (QM) is adopted, then this knowledge update is a passage from the description of all experimental data to the probabilistic description of some particular subsamples of the data. In principle, QBism (see Fuchs et al. [32]) also leads to rejection of speculations on instantaneous influence. In QBism knowledge update is related to subjective probability. However, in this paper we shall not appeal to QBism (cf. [33]).

This paper was also stimulated by the recent works of Plotnitsky [34,35] who analyzed quantum nonlocality in the framework of the original EPR presentation and the debate between Einstein and Bohr [21,36]. He operated with the notion of Einstein’s nonlocality which is similar to the notion of Lüders’ projection nonlocality. In any event, Einstein’s nonlocality is rooted in the Lüders projection postulate. Plotnitsky concluded that spooky action at a distance is in fact *“spooky predictions at a distance”*. I disagree with this terminology and I shall explain later why such terminology is also misleading (Section 7).

We start considerations with the extended citation from the practically unknown preprint of A. Aspect [5] (see also [4]). In this preprint, “projection nonlocality” came in all its brilliance.

## 2. Alain Aspect: Counter-Intuitiveness of Quantum Formalism

”Let us consider the optical variant of the Bohm’s version of the EPR Gedankenexperiment. A source *S* emits a pair of photons with different frequencies $\nu_{1}$ and $\nu_{2}$, counterpropagating along Oz. Suppose that the polarization part of the state vector describing the pair is:

$$|\Psi \left(\nu_{1},\nu_{2}\right)\rangle =\frac{1}{\sqrt{2}}\{|x,x\rangle +|y,y\rangle \},$$

where *x* and *y* are linear polarizations states.”

“Let us now consider the probabilities $p_{\pm \pm }\left(a,b\right)$ of joint detections of $\nu_{1}$ and $\nu_{2}$ in the channels + or − of polarisers *I* or *II* in orientations *a* and *b*. Quantum mechanics predicts:

$$p_{++}\left(a,b\right)=p_{--}\left(a,b\right)=\frac{1}{2}\cos^{2}\left(a,b\right),\;p_{+-}\left(a,b\right)=p_{-+}\left(a,b\right)=\frac{1}{2}\sin^{2}\left(a,b\right)$$

We are going to show that these quantum mechanical predictions have far reaching consequences.”

“As a naive physicist, I like to raise the question of finding a simple image to understand these strong correlations. The most natural way to find an image may seem to follow the quantum mechanical calculations leading to $p_{\pm ,\pm }\left(a,b\right).$ In fact, there are several ways to do this calculation. A very direct one is to project the state vector $|\Psi (\nu_{1},\nu_{2})\rangle$ onto the eigenvector corresponding to the relevant result. This gives immediately the joint probabilities $p_{\pm ,\pm }\left(a,b\right).$ However, since this calculation bears on state vectors describing globally the two photons, I do not know how to build a picture in our ordinary space.”

“In order to overcome this problem, and to identify separately the two measurements happening on both ends of the experiment, we can split the joint measurement in two steps. Suppose for instance that the measurement on photon $\nu_{1}$ takes place first, and gives the result (+), with the polarizer *I* in orientation *a*. The + result (associated with the polarization state |a〉) has a probability of 1/2. To proceed with the calculation, we must then use the postulate of reduction of the state vector, which states that after this measurement, the new state vector $|\Psi^{\prime }\left(\nu_{1},\nu_{2}\right)\rangle$ describing the pair is obtained by projection of the initial state vector $|\Psi (\nu_{1},\nu_{2})\rangle$ (Equation (1)) onto the eigenspace associated to the result +: this two dimensional eigenspace has a basis {|a,x〉,|a,y〉}. Using the corresponding projector, we find after a little algebra

$$|\Psi^{\prime }\left(\nu_{1},\nu_{2}\right)\rangle =|a,a\rangle .$$

This means that immediately after the first measurement, photon $\nu_{1}$ takes the polarization *a*: this is obvious because it has been measured with a polarizer oriented along *a*, and the result + has been found. More surprisingly, the distant photon $\nu_{2}$, which has not yet interacted with any polarizer, has also been projected into the state *a* with a well defined polarization, parallel to the one found for photon $\nu_{1}.$ This surprising conclusion, however, leads to the correct final result (3), since a straightforward application of the Malus law shows that a subsequent measurement performed along *b* on photon $\nu_{2}$ will lead to

$$p_{++}\left(a,b\right)=\frac{1}{2}\cos^{2}\left(a,b\right).\left(8\right)$$

The calculation in two steps, therefore, gives the same result as the direct calculation. But in addition, it suggests a picture for the two-step measurement:

- (i) Photon $\nu_{1}$, which does not have a well defined polarization before its measurement, takes the polarization associated to the obtained result, at the moment of its measurement: this is not surprising.
- (ii) When the measurement on $\nu_{1}$ is done, photon $\nu_{2},$ which does not have a well defined polarization before this measurement, is projected into a state of polarization parallel to the result of the measurement on $\nu_{1}.$ This is very surprising, because this change in the description of $\nu_{2}$ happens instantaneously, whatever the distance between $\nu_{1}$ and $\nu_{2}$ at the moment of the first measurement.”

“This picture seems in contradiction with relativity. According to Einstein, what happens in a given region of space-time cannot be influenced by an event happening in a region of space-time that is separated by a space like interval. It is therefore not unreasonable to try to find more acceptable pictures for “understanding” the EPR correlations. It is such a picture that we consider now.”

The latter is the hidden variable picture that led Bell to subquantum nonlocality which we call in his name *Bell nonlocality.*

Our aim is to show that the above picture does not contradict relativity. Moreover, it is schematically identical to the corresponding picture from classical probability theory: the probability-transformation resulting from conditioning on the concrete observation-output.

## 3. Lüders Nonlocality

In [8], Lüders formalized in the form of a postulate, the operation of the quantum state-transformation resulting from measurement with the concrete output, as a projection on the corresponding subspace of the state space. In fact, this postulate was actively used from the first days of quantum theory; e.g., in the EPR-paper [21]. Often misleadingly, the projection postulate is associated with the name of von Neumann with reference to his book [22]; often people even say that about the von Neumann projection postulate.

### 3.1. Lüders’ Quantum State-Transformation Resulting from Observations

In the original quantum formalism, an observable *A* is represented by a Hermitian operator acting in Hilbert state space *H*,

(1) $$\hat{A}=\sum_{x}x\;{\hat{E}}^{A}\left(x\right),$$

where $E^{A}\left(x\right)$ is the orthogonal projector on the subspace $H_{x}$ composed of eigenvectors with eigenvalue *x* (we consider only observables with discrete spectra).

For pure initial state |ψ〉, the post-measurement state is always again the pure state given by normalized projection:

(2) $$|\psi_{A=x}\rangle ={\hat{E}}^{A}\left(x\right)|\psi \rangle /∥{\hat{E}}^{A}\left(x\right)\;|\psi \rangle ∥.$$

Thus, measurement with output A=x induces the state-transformation:

(3) $$\left|\psi \rangle \to \right|\psi_{A=x}\rangle .$$

We also recall the Born rule for probability of *A*’s output:

(4) $$p\left(A=x;\psi \right)=∥{\hat{E}}^{A}{\left(x\right)\;|\psi \rangle ∥}^{2}.$$

At first glance, the projection transformation of the state given by (2) has nothing to do with nonlocality (neither the Born rule). However, by considering a compound system $S=(S_{1},S_{2})$, we shall obtain the state-transformation procedure making the impression of instantaneous action at a distance.

Let quantum state |Ψ〉 belong to tensor product $H=H_{1}⊗H_{2}$ of state spaces $H_{i}$ of systems $S_{i},i=1,2.$ Select some observable *A* on $S_{1};$ it is represented by Hermitian operator $\hat{A}$ given by (1), where ${\hat{E}}^{A}\left(x\right)$ acts in $H_{1}.$ This observable can be also treated as an observable on compound system S. The latter is represented by projector ${\hat{E}}^{A}\left(x\right)⊗I$ in H. By getting output A=x, we transform the state of *S* on the basis of Lüders projection postulate:

(5) $$|\Psi_{A=x}\rangle ={\hat{E}}^{A}\left(x\right)⊗I|\Psi \rangle /∥{\hat{E}}^{A}\left(x\right)⊗I|\Psi \rangle ∥.$$

Consider now an observable *B* on $S_{2}$ and its conditional measurement, under output A=x. By Born’s rule

(6) $$p\left(B=y|A=x;\Psi \right)\equiv p\left(B=y;\Psi_{A=x}\right)=∥{\hat{E}}^{B}\left(y\right)⊗I|\Psi_{A=x}{\rangle ∥}^{2}.$$

If state |Ψ〉 is separable, i.e., $\left|\Psi \rangle =\right|\psi^{\left(1\right)}\rangle ⊗|\psi^{\left(2\right)}\rangle ,$ then

(7) $$p\left(B=y|A=x;\Psi \right)=\frac{∥{\hat{E}}^{B}\left(y\right)⊗{\hat{E}}^{A}{\left(x\right)|\Psi \rangle ∥}^{2}}{∥{\hat{E}}^{A}\left(x\right)|\psi^{\left(1\right)}{\rangle ∥}^{2}}=∥{\hat{E}}^{B}\left(y\right)|\psi^{\left(2\right)}{\rangle ∥}^{2}.$$

Thus, for such a state, measurement of observable *A* on $S_{1}$ does not change statistics for measurements of observable *B* on $S_{2},$

(8) p(B=y|A=x;Ψ)=p(B=y;Ψ)

However, if a state is entangled, then generally

(9) p(B=y|A=x;Ψ)≠p(B=y;Ψ)

We remark that any state of *S* determines the states of its subsystems $S_{i},i=1,2;$ for the initial state |Ψ〉,

(10) $$\rho^{\left(1\right)}=Tr_{H_{2}}|\Psi \rangle \langle \Psi |,\;\rho^{\left(2\right)}=Tr_{H_{1}}|\Psi \rangle \langle \Psi |,$$

and, for the post-measurement state $|\Psi_{A=x}\rangle ,$

(11) $$\rho_{A=x}^{\left(1\right)}=Tr_{H_{2}}|\Psi_{A=x}\rangle \langle \Psi_{A=x}|,\;\rho_{A=x}^{\left(2\right)}=Tr_{H_{1}}|\Psi_{A=x}\rangle \langle \Psi_{A=x}|.$$

The above considerations can be represented in the form of probabilities with respect to the states of $S_{2}.$ For separable state |Ψ〉,

(12) $$\rho^{\left(2\right)}=\rho_{A=x}^{\left(2\right)}\;\mathrm{and}\;p\left(B=y;\rho^{\left(2\right)}\right)=p\left(B=y;\rho_{A=x}^{\left(2\right)}\right),$$

and, for entangled states, generally

(13) $$\rho^{\left(2\right)}\neq \rho_{A=x}^{\left(2\right)}\;\mathrm{and}\;p\left(B=y;\rho^{\left(2\right)}\right)\neq p\left(B=y;\rho_{A=x}^{\left(2\right)}\right),$$

Above formulas are just mathematical expressions. To have some physical picture, we should present their interpretation. The main issue (and problem) is a state’s interpretation. Nowadays, some experts in quantum foundations claim that all interpretations are equally useful; so it is meaningless to struggle for a “right interpretation”; others claim that it is even possible to proceed without an interpretation at all. I do not think so.

### 3.2. Statistical vs. Individual Interpretations of a Quantum State

The statistical interpretation of a quantum state is commonly associated with the names of Einstein and Ballentine [37].

**SI** *Quantum state* |ψ〉 *represents the statistical features of a large ensemble of identically prepared quantum systems.*

Therefore, a quantum state is not the “personal state” of an individual quantum system; say, that of an electron.

**II** *Quantum state* |ψ〉 *is the physical state of the individual quantum system.*

The individual interpretation of a quantum state was originally used by the majority of the quantum community. It is very often even coupled to the Copenhagen interpretation. However, we have to be careful when talking about the Copenhagen interpretation. (One should rather talk about interpretations in the spirit of Copenhagen; see Plotnitsky [38,39].) Von Neumann definitely used the individual interpretation [22], but not Bohr [40].

In particular, von Neumann considered the Schrödinger equation as describing the dynamics of the physical state of an individual quantum system, similarly to the Newtonian or Hamiltonian dynamics of a classical system.

### 3.3. Individual Interpretation: State’s Conditioning Transformation Resulting from Observations

If one accepts the individual interpretation, then according to the Lüders projection postulate, a measurement performed on a system $S_{1}$ may change, instantaneously, a physical state of $S_{2}$ in a distant location. This is apparent quantum nonlocality.

For example, consider two physical systems $S_{1}$ and $S_{2}$ prepared in a state:

(14) $$|\Psi \rangle =(|01\rangle +\left|10\rangle \right)/\sqrt{2},$$

where the vectors labeled as |0〉,|1〉 are eignevectors of $\hat{A}:H_{1}\to H_{1}$ and $\hat{B}:H_{2}\to H_{2},$ and both $H_{i}$ are qubit spaces. (We omit the indexes for these vectors, i.e., they should be ${|0\rangle }_{i}{,|1\rangle }_{i},i=1,2.)$ By measuring observable *A* on $S_{1}$ and getting output A=0, we found the compound system in the state $|\Psi_{A=0}\rangle =|01\rangle .$ Thus, according to the individual interpretation, the state of $S_{2}$ instantaneously becomes |ϕ〉=|1〉. Since by the individual interpretation a quantum state has the meaning of the state of the individual system, this is nothing other than action at a distance.

The incorrect interpretation of a quantum state, namely, the individual interpretation, is source of the apparent quantum nonlocality.

However, this is just a theoretical problem, as can be seen in the next section.

### 3.4. In the Laboratory: Emergence of Individual and Statistical Interpretations

This is the good place to stress that even those who use the individual interpretation of a quantum state understand well that all quantum predictions are of statistical nature. One can speak as much as one wants about the wave function of the individual electron, but in a laboratory he would collect statistics. The Born rule is applied to calculate the frequencies of experiment’s outcomes for an ensemble of quantum systems. In such calculations, quantum state ∥Ψ〉 represents statistical features of this ensemble (in the operational approach, ∥Ψ〉 represents some preparation procedure).

For example, von Neumann [22] consistently used the individual interpretation, but at the same time he pointed that experimental verification is possible only through von Mises frequency approach to probability. In the laboratory, von Neumann would use the statistical interpretation of QM. Thus, although by using the individual interpretation experimentalist confronts action at a distance—at the level of the theoretical consideration, at the experimental level he is in the same situation as one using **SI**.

Independently of an interpretation of the quantum state, the essence of the problem is in the comment presented in the introduction. (As I know from private conversations, its author uses the individual interpretation.) We are interested in its following part:

“But the empirical fact remains, that setting a measurement operator on site A has an instantaneous ’influence’ o site B, if you later calculate conditional statistics between the data of site A and site B. Additionally, this faster-than-light change of statistical correlation is already empirically proven.”

## 4. Aspect versus EPR Presentations

The reader can see that in [5] Aspect essentially followed the original EPR-reasoning [21]. However, he excluded the most questionable component of the EPR-reasoning—the elements of reality.

The main problem of both presentations is the absence of the explicit statement on the interpretation of a quantum state, neither Einstein, Podolsky, and Rosen, nor Aspect started with its identification. This made their reasoning fuzzy and generated misinterpretations.

This is the good place to remark that one of the problems of the quantum community is that “one has to understand” (typically through long conversations) what kind of state’s interpretation is used by another. Debaters do not declare their interpretations from the very beginning. I can only dream for a conference where everybody would have on his conference badge not only the affiliation, but also his interpretation of the wave function; say the individual or statistical. It would be much easier to understand what people mean by their statements. If somebody thinks that he can proceed without assigning any interpretation to the wave function, then this should also be reflected on the badge. However, maybe even the interpretation-badges would not help. Recently the reasoning that projection ⇒ nonlocality was presented to me by one of the top experts in the many-worlds interpretation of quantum mechanics.

Aspect uses the individual interpretation (as I know from the private conversations) and by treating a quantum state as a state of an individual photon, he confronts with projection nonlocality (Section 3.3). However, this is mathematical nonlocality. In the laboratory, everybody has to collect statistical data; i.e., use the statistical interpretation (Section 3.4).

We read in the paper [5]: *“This is very surprising, because this change in the description of* $\nu_{2}$ *happens instantaneously.”* Therefore, Aspect pointed to the instantaneous change of the description. However, why is this change in theoretical description surprising? In fact, De Broglie [41] was surprised by such surprise.

The interpretational basis of this surprise and the consequent belief in quantum nonlocality is the mixture of the theoretical use of the individual interpretation with the laboratory-use of **SI**. The reality of laboratory-collected statistical data creates the illusion of the reality of a quantum state. It seems that this quantum state reality fallacy has its origin in von Neumann book [22] (see Section 3.4).

We remark that *the EPR-paper was in fact directed against the individual interpretation.* This is a good place to mention the Einstein-Bohr debate [21,36]. (See also [26,27,28,34,35,38,39,42,43,44,45] for discussions that are relevant to the content of the present paper.) It seems that Einstein emphasized troubles of quantum mechanics induced by the individual interpretation. Bohr replied to Einstein in the spirit of **SI**, and by using the latter, Bohr could not recognize the problem that was declared by Einstein: incompleteness of quantum mechanics.

For our presentation, it is important that in the EPR-paper, measurements were conditional, the first measurement on $S_{1}$ and then the selection of some measurement on $S_{2}.$ Therefore, corresponding probabilities are also conditional probabilities. Hence, by treating the EPR-Bohm probabilities as conditional, Aspect followed the EPR-paper.

In fact, the conditional probability picture reflects properly the context of experiments testing violation of the Bell type inequalities. The joint measurement picture is used too straightforwardly. In real measurements, photons in Lab 1 and Lab 2 are not detected simultaneously. (Therefore, the time window should be introduced.) Thus, this is really the conditional measurement, first for photon $\nu_{1}$ and then for photon $\nu_{2},$ or vice versa.

One may say that in Bell tests, one uses time windows only to create a spreadsheet containing paired data of *A* and *B*, and at this stage an order of the measurements does not exist. However, in a real experiment the probability that instances of detection of *A* and *B* coincide equals zero. It is always either $t_{A}<t_{B}$ or vice verse.

Moreover, even theoretically, measurements in the EPR-Bohm experiments cannot be treated as joint measurements. Consider the von Neumann scheme [22] for joint measurement of two compatible observables, say *A* and B, with Hermitian operators $\hat{A}$ and $\hat{B}.$ To measure *A* jointly with B, one has to represent them as functions of the same observable, say C, described by operator $\hat{C}$ with nondegenerate spectrum, in terms of operators $\hat{A}=f\left(\hat{C}\right),\hat{B}=g\left(\hat{C}\right).$ However, in the EPR-Bohm framework this observable *C* is nonlocal—in the usual sense, its measurement involves measurements in both laboratories (see [46] for discussion; the same viewpoint was presented in [47,48]).

## 5. Classical Probability: State’s Conditioning Transformation Resulting from Observations

Consider now classical probability theory (CP). It was mathematically formalized in set and measure theoretical framework by Kolmogorov in 1933 [49]. Let Ω be a set (of any origin) and let *P* be a probability measure on it. (To be mathematically rigorous, we should also consider a collection of subsets F of Ω forming Boolean (σ-)algebra. Measure *P* is defined for sets from F. If Ω is finite, then F is collection of all its subsets.)

A random variables is map A:Ω→R having some special property (measurability). We shall consider only random variables with the discrete range of values. For *A* and its value x, set $\Omega_{A=x}=\left\{s\in \Omega :A\left(s\right)=x\right\}$ and define its probability distribution as $p_{A}\left(x\right)=P\left(\Omega_{A=x}\right).$

In applications of CP to statistical physics set Ω describes an ensemble of systems (physical or virtual). Generally set Ω is infinite. These applications are based on the following mathematical model for states and observables:

- States are represented by probability measures;
- Observables are represented by random variables.

Here states are not states of individual systems, but of ensembles of systems (so to say statistical states). A state, probability measure P, represents a preparation context determining (statistically) features of systems belonging to Ω. It is useful to invent the following terminology: a *random systemS* is ensemble Ω. Then, probability measures represents the states of a random system. Denote its state space by $P\equiv P_{S}.$ Finally, we recall that ensembles need not be physical; they can be virtual as in the Gibbs considerations (see Schrödinger’s excellent book [50] on the latter approach).

Let A,B be random variables (representing classical observables) with probability distributions $p_{A}\left(x\right),p_{B}\left(y\right)$ and conditional probability p(B=y|A=x). We recall that the latter is given by *the Bayes formula:*

(15) p(B=y|A=x)=P(B=y,A=x)/P(A=x),

for P(A=x)>0. It should be emphasized that in CP the Bayes formula is the *definition* of conditional probability. It is not a theorem, it cannot be derived from other “natural postulates” (cf. the projection postulate in QM.) This formula can be represented in the form

(16) $$p\left(B=y|A=x\right)=P\left(\Omega_{B=y}\cap \Omega_{A=x}\right)/P\left(\Omega_{A=x}\right).$$

From the ensemble viewpoint, this formula means that we select subensemble $\Omega_{A=x}$ consisting of systems that generate outputs s→A(s)=x and then find proportion (with respect to the weight-measure P) of its elements that generate the output s→B(s)=y. It is useful to introduce for each value A=x the conditional probability measure $P_{A=x}:$

(17) $$P_{A=x}\left(E\right)=P\left(E\cap \Omega_{A=x}\right)/P\left(\Omega_{A=x}\right).$$

Then $p\left(B=y|A=x\right)=P_{A=x}\left(B=y\right).$ To make classical and quantum notations consistent, we set

(18) $$p_{A}\left(x\right)=p\left(A=x;P\right),p_{B}\left(y\right)=p\left(B=y;P\right),\;p\left(B=y|A=x\right)=P\left(B=y;P_{A=x}\right).$$

Consider a random system *S* in state P; perform *A*-observations and select those with the concrete output A=x. By using the Bayes formula we obtain the new state of *S* given by conditional probability measure $P_{A=x}.$ The conditioning transformation in state space P (the space of probability measures)

(19) $$P\to P_{A=x}.$$

is the direct analog of the conditioning transformation in quantum state space given by the Lüders projection postulate, see (3), resulting from *A*-measurement. This transformation changes the state of random system *S*, and, for any observable (random variable) B, its probability distribution is modified:

(20) $$p\left(B=y;P\right)\to P\left(B=y;P_{A=x}\right).$$

Now, consider a compound random system $S=(S_{1},S_{2}),$ with random subsystems $S_{i},i=1,2$ given by ensembles $\Omega_{i}.$ Then *S* is given by ensemble $\Omega =\Omega_{1}\times \Omega_{2}.$

If random systems are *independent,* then the state of *S* is given by $P=P^{\left(1\right)}⊗P^{\left(2\right)},$ where $P^{i},i=1,2,$ are are states of $S_{i}$ and the probability measure *P* is defined as $P\left(E_{1}\times E_{2}\right)=P^{\left(1\right)}\left(E_{1}\right)P^{\left(2\right)}\left(E_{2}\right).$ We call such probability state *separable.* Separability is just another term for independence of random variables $I_{i}:\Omega \to \Omega_{i},I_{i}\left(s\right)=s_{i}.$ If probability measure *P* on Ω is not separable, we call this state *entangled.*

Let *A* and *B* be observables on random systems $S_{1}$ and $S_{2}$ given by random variables, $A:\Omega_{1}\to R$ and $B:\Omega_{2}\to R.$ They can be treated as observables on *S* represented by random variables $A\left(s\right)=A\left(s_{1}\right),B\left(s\right)=B\left(s_{2}\right).$ It is easy to see that for a separable state $P=P^{\left(1\right)}⊗P^{\left(2\right)},$

(21) $$P\left(B=y;P_{A=x}\right)=p\left(B=y;P\right);$$

cf. the quantum case, (8). Therefore, the state transformation of induced by requiring that output A=x does not change probability distribution for *B*-observable. However, if *P* is entangled, then generally the probability distribution of *B* is modified:

(22) $$P\left(B=y;P_{A=x}\right)\neq p\left(B=y;P\right);$$

cf. the quantum case, (9).

We remark that, similarly to the quantum case, in the classical case state *P* of *S* determines states of $S_{i},$ as the marginal probabilities

(23) $$P^{\left(1\right)}\left(E_{1}\right)=\int_{E_{1}\times \Omega_{2}}dP,\;P^{\left(2\right)}\left(E_{2}\right)=\int_{\Omega_{1}\times E_{2}}dP,$$

for discrete parameter-spaces, integration is reduced to summation. If a state is separable, $P=P^{\left(1\right)}⊗P^{\left(2\right)},$ then its marginals coincide with $P^{\left(1\right)},P^{\left(2\right)}.$ Hence,

(24) $$P_{A=x}^{\left(2\right)}=P^{\left(2\right)}\;\mathrm{and}\;P\left(B=y;P_{A=x}^{\left(2\right)}\right)=p\left(B=y;P^{\left(2\right)}\right),$$

where $P_{A=x}^{\left(2\right)}$ is the marginal of the transformed state $P_{A=x};$ cf. the quantum case, (12). For entangled state P, this equality is violated. Generally

(25) $$P_{A=x}^{\left(2\right)}\neq P^{\left(2\right)}\;\mathrm{and}\;P\left(B=y;P_{A=x}^{\left(2\right)}\right)\neq p\left(B=y;P^{\left(2\right)}\right);$$

cf. the quantum case, (13).

If we operate with theoretical probabilities, then the conditioning transformation is practically instantaneous. In any event, the time of calculating probabilities does not depend on the distance between $S_{1}$ and $S_{2}.$ This also matches well to operating with virtual ensembles in the Gibbs approach. However, the empirical reconstruction of transformed state $P_{A=x}$ takes the time needed to collect the statistics of A=x outputs.

The above scheme of conditional observations was based on the statistical interpretation of probability. We briefly describe this scheme from the viewpoint of subjective probability. Here state transition (19) really happens practically instantaneously, but in the information space (not in the physical space). Additionally, the users of subjective probability do not speak about action at a distance. Everyone understands well that subjective probability theory is about predictions (including “predictions at a distance”; cf. [34,35]).

## 6. Statistical Interpretation: A State’s Conditioning Transformation Resulting from Observations

In Section 3.1 we presented the mathematical scheme of the conditioning transformation of a quantum state resulting from observations. Now it will treated within the statistical interpretation of QM with the emphasis of the analogy with the CP-scheme (within the statistical approach to probability). In Section 3.1 we operated with pure states, since, for the individual interpretation, nonlocality of the Lüders type is straightforwardly associated with conditioning transformations of pure states. For the statistical interpretation, it is natural to proceed with so-called mixed states represented by density operators. Denote the space of density operators by the symbol D. By the statistical interpretation a quantum state (density operator) describes statistical features of an ensemble of quantum systems corresponding to some preparation context—a *quantum random system.* We remark that this interpretation is applicable even to pure quantum states, density operators given by one dimensional projectors. Generally the terminology “mixed state” is misleading, at least for the statistical interpretation. In this framework the Lüders projection postulate loses its special role. In principle, one can consider any (affine) transformation of *D* as a conditioning transformation, as is done in theory of quantum instruments [23,24]. We remark that the latter is based on the statistical interpretation of quantum states.

Let ρ be the state of quantum random system *S* and let A,B be quantum observables. Their probability distributions are given by the Born’s rule:

(26) $$p\left(A=x;\rho \right)=TrE^{A}\left(x\right)\rho ,\;p\left(B=y;\rho \right)=TrE^{B}\left(y\right)\rho .$$

Measurement of quantum observable *A* with output A=x generates the conditioning transformation in space *D* of quantum states:

(27) $$\rho \to \rho_{A=x}=E^{A}\left(x\right)\rho E^{A}\left(x\right)/Tr\left(E^{A}\left(x\right)\rho E^{A}\left(x\right)\right).$$

In sequential *B*-observation the concrete output B=y is obtained with probability given by the Born’s rule for the transformed state $\rho_{A=x}.$ This process can be considered as transformation of probability distribution:

(28) $$p\left(B=y;P\right)\to p\left(B=y;\rho_{A=x}\right)=TrE^{B}\left(y\right)\rho_{A=x}.$$

By the statistical interpretation this mathematical formalism describes the following process:

- Ensemble Ω with statistical features described by state ρ is prepared;
- *A*-measurement is performed;
- Systems corresponding to output A=x are selected and new ensemble $\Omega_{A=x}$ is created;
- For systems belonging to $\Omega_{A=x},$*B*-observation is performed.

Of course, in the theoretical framework, transitions (27) and (28) can be considered as practically instantaneous. However, empirically the creation of ensemble $\Omega_{A=x}$ is not instantaneous at all. Therefore, in the statistical interpretation there is no place for spooky action at a distance.

Typically, by referring to the statistical interpretation one emphasizes the statistical interpretation of quantum states. We elevate the role of the state transformation $\rho \to \rho_{A=x}$ and the corresponding probability transformation $p\left(B=y;P\right)\to p\left(B=y;\rho_{A=x}\right).$ Additionally, the former is considered as just mathematical representation for the latter. *QM is a special theory of probability transformations.* This is the essence of *the Växjö interpretation* [31].

We also point to QBism, the subjective probability interpretation of quantum mechanics. QBists’ position is the same as classical subjectivists: “Faster-than-light change of statistical correlation” is a mental process (well, even this process takes some time) [32]. QBism also treats QM is probability transformation theory. However, in contrast to the Växjö interpretation, QBism emphasizes that QM is generalization of the Bayesian inference procedure of CP endowed with subjective interpretation of probability. Of course, quantum Bayesian inference can used be for decision making (in particular, outside of physics [33]. However, it seems that quantum (Lüders) nonlocality debate has no direct relation to Bayesian inference, even from the subjective probability viewpoint. In fact, I have not seen publications on the QBism-based analysis of violation of the Bell type inequality.

The separate problem, i.e., having no relation to quantum nonlocality, is why CP and QM formalisms have different mathematical structures. Since this question has no direct relation to compound systems, we shall discuss it only briefly, in Appendix A.

## 7. “Spooky Prediction at a Distance?”

Consider a theoretical operation with spatially separated quantum observables; i.e., let us do mathematical calculations based on the Lüders projection postulate and the Born rule. We can evaluate the conditional marginal distribution for Bob outcomes which are paired with a particular Alice’s outcome, A=x. This is the distribution $p(B=y;\rho_{A=x})$; see (28). Plotnitsky [34,35] called this evaluation *“spooky prediction at a distance”*. It seems that the term “prediction” is not appropriate for such an evaluation. In probability theory, prediction is typically associated with Bayesian inference. However, the explicit conditioning-transformation given by (28) is not inference. Nevertheless, in papers [34,35] the prediction-terminology can be justified as related to the ERP elements of reality. However, even within the EPR-framework referring to “spookiness” is misleading.

By Plotnitsky, “spookiness” of quantum predictions is due to the impossibility to present any space-time picture explaining correlations. We shall criticize this viewpoint in Section 8.

## 8. The Hertz-Boltzmann Viewpoint on Creation of Scientific Theories

By criticizing Plotnitsky, I again (cf. [51,52]) refer to the Hertz-Boltzmann [53,54,55,56] methodology of scientific theories. By this methodology, there are two levels of scientific representation of natural phenomena:

- A theoretical model (TM);
- An experimental model (EM).

A TM respects the universal *law of cause and effect.* It states that for every effect there is a definite cause; likewise, for every cause, there is a definite effect. EM provides a consistent description and prediction for experimental data. As early as in the 19th century, scientists understood well (at least Hertz and following him Boltzmann) that experimental data is statistical and its description and theoretical prediction are probabilistic. For them, it was clear that EMs need not be causal (cf. von Neumann [22], acausality of quantum measurements). Of course, TM and EM have to be coupled, TM→EM. However, coupling is not rigid, a TM is not rigidly coupled to experiments; a TM is our mental image (“Bild”) of physical phenomena; its main aim is to respect the law of cause and effect. In short, Hertz and Boltzmann, by developing the “Bild-concept,” were precursors of Bell with his attempt to introduce hidden variables in quantum theory. The main difference between the approaches of Hertz-Boltzmann and Bell is that Bell proposed the very rigid rule connecting TM and EM (in this case EM = QM). He wanted TM to describe the concrete outputs of quantum measurements:

A=A(λ).

Here λ, a hidden variable, is an element of TM; the right-hand *A* is also in TM, but the left-hand *A* is in EM.

It is interesting that Bell was well aware of the problem of coupling of TM and EM (=QM). He started his activity [7] with a really strong critique of von Neumann’s no-go theorem [22]. Bell criticized precisely too rigid coupling TM→EM in von Neumann’s consideration. In fact, all no-go statements are just statements about selection of possible TM for QM and the correspondence rule, TM→EM. Unfortunately, creators of QM were not aware about the “Bild-concept” of Hertz-Boltzmann (or just ignored it? Schrödinger tried to appeal to it, but his message was completely ignored).

Coming back to the Einstein-Bohr debate, we can say that Einstein said that QM is not a TM, Bohr replied that he did not see a problem, since he knows that QM is a EM. It seems that Bohr did not reject a possibility to construct a consistent TM for QM (treated as EM), but he would not accept the Hertz-Boltzmann-Schrödinger viewpoint on the structure of a scientific theory. He considered such kind of activity as metaphysical, and hence, meaningless. In contrast, Einstein badly wanted a TM for QM, but (as latter Bell) he wanted too much from the map TM→EM.

As to one of possible TMs for EM = QM, we can point to *prequantum classical statistical field theory* (PCSFT) [57], the classical random field model. Coupling of TM = PCSFT with EM = QM is very simple; a quantum state (density operator) is identified with the covariance operator of a complex random field that is normalized by the field energy; a quantum observable (Hermitian operator) corresponds to a quadratic form of a field.

Did Bohr and Einstein (as well as, say, Heisenberg and von Neumann) know about the works of Hertz and Boltzmann [53,54,55,56]? The situation is really strange, since all mentioned fathers of QM could read in German, and Hertz and Boltzmann were very famous.

Finally, we remark that modern philosophers operate with a similar scheme of the two-level structure of scientific theories [58]:

- Ontic;
- Epistemic.

It is surprising that philosophers (who really read a lot) are not aware of the works of Hertz and Boltzmann. However, this not the main problem with the ontic-epistemic approach. The main problem is that the ontic level represents reality “as it is” (when nobody makes measurements). For Hertz and Boltzmann, TM was not about reality as it is, but just its mental “Bild” being consistent and respecting the law of cause and effect.

Now, turn to “spooky predictions”. We recall that “spookiness” is related to the impossibility to construct a causal picture of physical phenomena. However, generally it is not EM’s aim to present a causal picture. The latter is given within TM. (Once again, QM ≠ TM, QM = EM.) Therefore, I think that Plotnitsky’s slogan “spooky prediction at a distance” is misleading, no different to Einstein’s slogan “spooky action at a distance”.

## 9. Probability Conditioning: Quantum versus Classical Optics

Classical optical correlations can reproduce the quantum correlations of the EPR-Bohm experiment (see, e.g., [29,59,60,61,62,63]). Hence, such classical correlations violate, e.g., CHSH-inequality. Moreover, classical optics can be represented in the quantum-like manner, i.e., within complex Hilbert state space, as was done in works on so-called classical entanglement [59,60,61,62,63]. This shows that the devil is not in the states, but in measurement procedures. The corresponding detailed analysis was presented in the author’s paper [3]. The crucial difference between measurement procedures in quantum and classical optics is the discrete structure of outputs of quantum measurements, in contrast to continuous measurements of intensity in classical optics. This difference, discrete detection events versus continuous intensity, was emphasized by Bohr and formalized in the notion of phenomena [34,35,38,39,40]; e.g., a dot on the photo-emulsion screen in the two slit experiment. This difference is reflected even at the state level in the notion of quantum superposition as the superposition of two discrete events; say, the detection of spin-up or spin-down (see Dirac [64] and recent paper [3], including the corresponding citation of Dirac; see also Jaeger [42]). The crucial point is that superposition’s meaning is coupled to the possible outcomes of observations; before measurement a system is in the superposition of its possible outcomes.

The discrete structure of detection events is the essence of objectification of quantum physics (cf. Jaeger [43]), through association of outputs of say photo-detectors with photons. (“What is photon?” “It is detector’s click!”—from my conversation with A. Zeilinger.) This philosophical issue is coupled to experiments, via experiments of Grangier’s type on coincidence detection for outputs of a beam splitter (see, e.g., [65]).

Thus, although at the formal mathematical level the state conditioning in classical optics coincides with quantum state conditioning, their probabilistic meanings are different. The latter (quantum) formalizes probability conditioning based on statistics of discrete events and it is close to classical probability conditioning based on the statistical interpretation of probability.

This is a good place to remark that nowadays the aforementioned crucial difference between quantum and classical measurement procedures is commonly neglected, even in foundational works (maybe besides the works of Plotnitsky who permanently emphasized the role of Bohr’s notion of phenomena in quantum foundations). Personally, I understood the importance of Bohr’s phenomena for quantum foundations only by analyzing the difference between quantum and classical entanglements [3] (which is in fact reduced to difference between quantum and classical superpositions). In particular, this issue is typically neglected in the statistical interpretation (cf. works on reformulation of quantum foundations of the basis of von Mises’s frequency probability theory [12]). Additionally, in the Växjö interpretation it has to be stressed that quantum formalism describes the machinery for probability conditioning based on outcomes of *discrete observations* (generating quantum phenomena).

## 10. Concluding Remarks

The aim of this paper is the disillusion of projection-based nonlocality (Lüders nonlocality). This sort of nonlocality can be considered as apparent quantum nonlocality, in contrast to subquantum (Bell equality based) nonlocality. It is important to distinguish these two nonlocalities sharply. They are often mixed in the heads of scientists advertising “quantum nonlocality.” This two-faced Janus is often seen as having just one face—quantum nonlocality.

This mental mixing is explainable by taking into account coupling between Lüders and Bell nonlocalities. This coupling was excellently presented in Aspect’s paper [5]. According to him, Lüders nonlocality makes quantum theory so counter-intuitive that any common, sensible scientist would try to find a beyond-quantum explanation.

Bell proposed a class of subquantum models known as models with hidden variables [6,7]. For such models, he derived the inequality and its violation was interpreted by him as the evidence of another sort of nonlocality (Bell nonlocality). Bell nonlocality has no precise meaning. Its invention is based on Bell’s unjustified claim that if his inequalities are violated, then to explain quantum correlations there must exist instantaneous influences between distant measuring stations. This claim leads to something really amazing: *Bell nonlocality was elevated to quantum physics and also was treated as quantum nonlocality.* Two-faced Janus of quantum nolocality was born.

In [1], it was shown that in the purely quantum framework violation of the Bell type inequalities is a consequence of local incompatibility of observables (e.g., observables $A_{1}$ and $A_{2}$ on system $S_{1}).$ Thus, Berll nonlocality has nothing to do with quantum mechanics. This is a feature of one very special class of subquantum models considered by Bell [6,7].

In the present paper, it was shown that Lüders nonlocality is the typical “nonlocality” of probability conditioning, similar to “nonlocality” of CP-conditioning. Both faces of nonlocality-Janus were destructed.

In this paper, we emphasized the role of two basic interpretations of a quantum state, individual (physical) vs. statistical. Following Aspect’s reasoning and assuming that a quantum state is the physical state of the individual quantum system, one can really confront Lüders nonlocality. On the other hand, the statistical interpretation combined with treatment of quantum mechanics as machinery for probability conditioning (the Växjö interpretation [31]) implies that Lüders nonlocality is typical “nonlocality” of probability conditioning.

Coming back to Summhammer’s comment cited in introduction and his question, I say that the right scientifically-justified terminology for “faster-than-light change of statistical correlation” is *probability conditioning on the basis of the quantum calculus.* (We stress, see Appendix A, that the latter is the probability calculus designed for operating with incompatible observables.) If the statistical interpretation of QM is adopted, then this probability conditioning is a passage from the description of all experimental data to the probabilistic description of some particular subsamples of the data. There no place here to talk about the speed of changes because there is no notion of time related to such an update. 

## Appendix A. Probability Calculi for Compatible and Incompatible Observables

Here we emphasize the difference between classical and quantum probability calculi. First of all, we remark that for compatible observables, quantum probability calculus is reduced to classical. For compatible observables, it is possible to determine the joint probability distribution (jpd) that can be used to represent the state of a random system. Hence, the origin of the difference is in the existence of incompatible observables. In probabilistic terms (see [13]), *incompatibility of observables is equivalent to nonexistence of jpd.* The mathematical formalism of quantum mechanics gives a new rule for the probability conditioning resultion from observations. This rule is more general than the classical one. This is the good place to cite Feynman [66]:

“From about the beginning of the twentieth century, experimental physics amassed an impressive array of strange phenomena which demonstrated the inadequacy of classical physics. The attempts to discover a theoretical structure for the new phenomena led at first to a confusion in which it appeared that light, and electrons, sometimes behaved like waves and sometimes like particles. This apparent inconsistency was completely resolved in 1926 and 1927 in the theory called quantum mechanics. The new theory asserts that there are experiments for which the exact outcome is fundamentally unpredictable, and that in these cases one has to be satisfied with computing probabilities of various outcomes. But far more fundamental was the discovery that in nature the laws of combining probabilities were not those of the classical probability theory of Laplace.”

In particular, the quantum calculus of probabilities for incompatible observables, i.e., those without jpd, generates the EPR-Bohm(-Bell) correlations. It is commonly emphasized that these correlations are *nonlocal.* It is important to assign the explicit meaning to this terminology. The plane meaning is “correlations between outcomes of measurements performed for spatially separated systems.” In this sense CP-correlations for spatially separated systems are also nonlocal. Of course, this meaning is not assigned to the nonlocality of EPR-Bohm(-Bell) correlations. Typically by speaking about nonlocal quantum correlations, one emphasizes their generation via spooky action at a distance. However, as was shown in [1], the crucial issue is the local incompatibility of observables; e.g., incompatibility of spin projections for system $S_{1}$ (or $S_{2}).$ Since these correlations are based on incompatible observables, they are nonclassical from the viewpoint of probability theory. They are not based on common jpd.

Finally, we remark that the quantum calculus of probability is a special calculus of *contextual probability* [13]. Here contextuality is understood as dependence of probability distribution on the experimental context. Thus, such contextuality is more general than contextuality coupled to joint measurement with a compatible observable, as is commonly considered in the Bell framework. The crucial point is that (in accordance with the Bohr complementarity principle) there exist incompatible experimental contexts that cannot be reduced to the common context.
